# Bone Density and Implant Primary Stability. A Study on Equine Bone Blocks

**DOI:** 10.3390/dj7030073

**Published:** 2019-07-01

**Authors:** Francesco Orlando, Federico Arosio, Paolo Arosio, Danilo Alessio Di Stefano

**Affiliations:** 1 Dental School, University of Milan, 20132 Milan, Italy; 2 Private Practice, 20871 Vimercate, Italy; 3 Dental School, Vita-Salute University and IRCCS San Raffaele, Via Olgettina 58, 20132 Milan, Italy

**Keywords:** bone density, implant stability, equine bone blocks

## Abstract

Previous results on synthetic blocks mimicking bone indicate that bone density can be measured by the friction encountered by a rotating probe while it descends into bone, and that primary implant stability may be measured through the integral (I) of the torque–depth curve at implant insertion. This study aims to repeat those tests on collagen-preserving equine bone blocks as they better reproduce the mechanical properties of natural bone. Fifteen cancellous equine blocks had their density measured using a measuring probe. This was compared to their known physical density through linear regression analysis. Implant placement was carried out into six cancellous equine blocks and primary stability was measured using (I), as well as the insertion torque (IT), the implant stability quotient (ISQ), and the reverse torque (RT). The relation between (I), (IT), (ISQ), and (RT) was investigated by correlation analysis. Bone density measured using the probe correlated significantly with actual density, both with (r = 0.764) and without irrigation (r = 0.977). (I) correlated significantly with IT and RT under all irrigation conditions, and with ISQ only without irrigation (r = 0.886). The results suggest that the probe provides actual bone density measurements. They also indicate that (I) measures primary implant stability and is more sensitive to density variations than IT, RT, and ISQ. Results are consistent with those obtained on synthetic blocks but suggest that equine bone blocks may better reproduce the mechanical properties of human cancellous alveolar bone. This should be the subject of additional studies.

## 1. Introduction

Bone density is one of the main factors determining implant stability [1,2]. This, in turn, determines successful osseointegration [3]. Its accurate measurement prior to implant placement may allow the surgeon to adjust site preparation and achieve appropriate stability to load the implant according to the best possible protocol [4]. The other factor influencing primary implant stability is the presence and thickness of a cortical bone layer that may further stabilize the implant because of its structural and mechanical properties [5,6,7,8]. Cortical bone, in fact, displays significantly different anatomical features than cancellous bone, namely no trabecular spaces, which are replaced by a dense matrix made of mineralized collagen fibers [8]. This gives cortical bone significantly different mechanical properties: cortical bone has a greater density, compressive strength and stiffness [7,8,9,10]. These properties show less intra- and intersubject variability than those of cancellous bone [11]. When a dense a thick cortical bone layer is present at the implant insertion site, according to the position in the jaw [12], it may be exploited by the oral surgeon to give the implant additional primary stability, especially when trabecular bone has a low density. The implant head, in fact, may engage the cortical bone layer to an extent that depends on the implant site underpreparation [13,14,15].

Recently, a micromotor featuring an instantaneous bone density measurement system has been introduced in the clinical practice. This system was shown to provide reliable density measurements in a series of studies involving bovine bone ribs [16] and polyurethane foam blocks [17]. On bovine ribs, bone density measurements carried out using the density measuring micromotor were shown to correlate significantly with histomorphometric bone density [16]. On foam blocks, the system was shown to provide reliable bone density measurements that were bound to density by a linear relation, which was regarded as the calibration curve of the system [17]. Yet, when measurements were carried out on foam blocks under irrigation, a condition better mimicking that found in the clinical setting, the calibration curve changed in a way that suggested that the liquid lubricating effect was dependent on block density, a result that was unexpected and called for further observations. The micromotor used in these studies also allows to measure implant primary stability by calculating the integral (I) of the torque–depth curve while the implant is being placed. Such a quantity, under the conditions that the implant threads are evenly spaced and the rotation speed is constant, is proportional to the insertion energy, which is the total amount of energy needed to place the implant into its site. This parameter, being dynamic, that is, being the result of all the interactions occurring between the implant and the surrounding bone while the implant is being placed, may convey more information on stability than standard stability parameters such as the insertion torque (IT) and the implant stability quotient (ISQ) that may be collected in the clinical setting [18,19,20]. Indeed, the torque–depth integral (I) was shown to correlate significantly to bone to implant contact area (BIC) in two studies on bovine ribs [21] and humans [22]. Understanding how it relates to bone density and to the macro- and micro-characteristics of the implant being used may therefore be pivotal to design patient- and implant-tailored insertion protocols allowing to carry out safely and effectively immediate loading rehabilitation or rehabilitation in low-density, low-quality bone sites [15,23]. This relation, at present, is still far from being understood. In a study on bovine bone, the insertion energy (which, as said, differs from (I) by a multiplicative constant only), was found to not correlate with IT and ISQ values [24] and in a study on foam blocks, the integral (I) was observed to be more sensitive to density variations than IT, ISQ, and reverse torque (RT) [25]. Still, integral–density curves, while linear in a subset of density values, showed an unexpected plateau at higher densities that was attributed to the features of the foam blocks. These unexplained observations possibly indicated that foam blocks, while having macroscopic properties (that is, density, resistance to compression, resistance to shear forces) overlapping those of bone [26], may not mimic appropriately some other bone properties like local elastic reactions [27]. Foam blocks, in fact, lack completely of a trabecular structure like that of bone. More reliable data might be provided by studies on fresh bovine ribs; yet, bovine rib segments are highly variable as far as bone density and the thickness of the cortical layer are concerned. This implies that reliable results from studies on bovine ribs may be achieved only carrying out a relatively high number of experiments and still they should be interpreted with care. An equine material has been placed on the market as a bone substitute to be used in dentistry, maxillofacial surgery and orthopedics [23,28,29,30,31,32,33,34,35,36]. Interestingly, the process equine bone is subjected to manufacture this graft is a mild, collagen-preserving one, implying that equine bone blocks achieved through this process retain their trabecular structure and their original mechanic characteristics. Further, as small cancellous blocks are achieved by sectioning large equine femurs, they may be regarded as homogeneous, as far as bone density is concerned, throughout their volume. These characteristics suggest that these equine blocks may better mimic human bone than foam ones, while being more homogeneous than bovine ribs, and may therefore be a reliable testing material. This study aims to investigate the relation between bone density, the integral of the torque–depth curve, and other classical primary stability measuring quantities in such bone model system. To the authors’ knowledge, this study differs from current published literature on the subject as it’s the first example of the use of such equine bone blocks in an in vitro study concerning bone density and primary implant stability measurement.

## 2. Materials and Methods 

### 2.1. Equine Cancellous Bone Blocks

**Figure 1:**
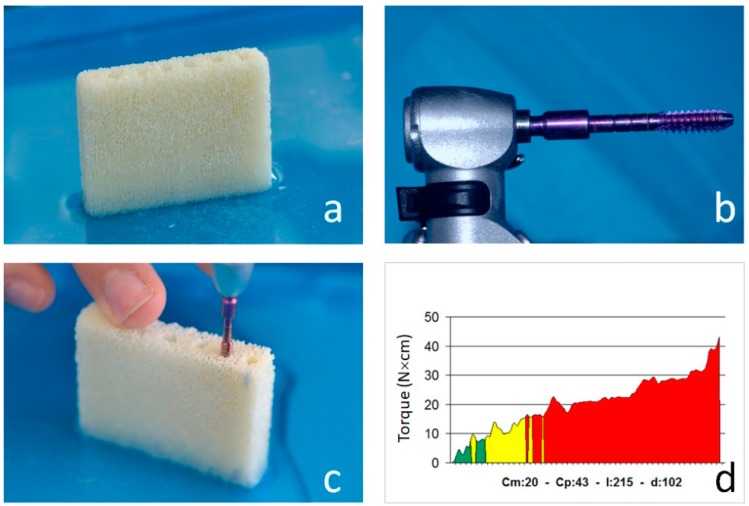
(**a**) One of the bone blocks used in the present study. (**b**) The bone density measuring probe. The probe is a 2-mm-wide cylinder featuring equally spaced threads whose width is mm and shape is a 1-degree reverse cone. When the probe screws into the hole (**c**), only the first thread exerts friction on the bone walls, because of the reverse cone shape. The micromotor display shows an instantaneous torque–depth curve (**d**), also indicating the average torque (Cm, N × cm), the peak torque (Cp, N × cm), the integral of the curve (I, N × cm^2^/100) and the depth (d) reached by the probe in tenths of millimeter.

Equine cancellous bone blocks used in the present study (Osteoplant, Bioteck, Arcugnano, Italy) were similar, but not identical, to the corresponding commercial bone grafts destined to be implanted in patients undergoing orthopedic surgeries. While dimensions were identical to those being marketed, their density varied in a wider range, the commercial ones having a density between 0.40 and 0.80 g/cm^3^ and those used in the present study having a density between 0.30 and 1.12 g/cm^3^. They were rectangular, their nominal dimensions being 10 × 10 × 30 (Bioteck code OSP-01C) and 10 × 10 × 50 mm (Bioteck code OSP-01E) (Figure 1a). Their actual dimensions—length (L), height (H), and thickness (D)—were calculated as the average of three measurements taken in three different positions (the two borders and the center) using a digital caliper (ABS Digimatic Caliper, Mitutoyo, Kawasaki, Japan). The block volume V was then calculated as V = L × H × T, the error being calculated using the formula for propagation of errors. Blocks were then weighted three times using a high precision scale (PCE-BSH-6000, PCE Instruments, Ensign Way, UK) and their weight W was calculated as the average of the three measurements. Their density (D) was then calculated using the formula D = W/V, and the corresponding error was calculated using again the formula for propagation of errors. Fifteen bone blocks were used.

### 2.2. Implants

Implants used in the present study were cylindrical and featured a double-etched, sandblasted surface (Stone, IDI Evolution, Concorezzo, Italy). Their threads are evenly spaced, with a known pitch, i.e., this implant meets the conditions implying that the torque–depth integral at placement and the insertion energy differ only by a multiplicative constant. Implants used in the present study were all sized 3.75 × 12 mm.

### 2.3. Instantaneous Torque Measuring Micromotor

The device was a TMM2 surgical micromotor (IDI Evolution) featuring an instantaneous torque measuring system. At implant insertion, the micromotor samples instantaneous torque (iT) at high frequency and records the depth the implant has reached. While the implant is being inserted, the device shows the (iT)/depth curve.

The micromotor also displays the average (Cm), the peak torque (Cp), also known as insertion torque (IT), and the torque/depth curve Integral (I) values (Figure 1d).

### 2.4. Experimental Procedure

**Figure 2:**
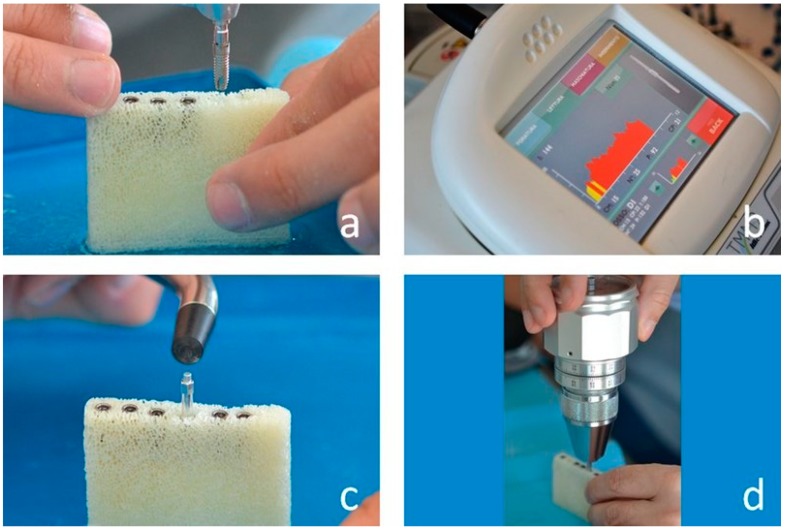
(**a**) One of the implants before insertion. Implants used in the present study were cylindrical 3.75 × 12 mm fixtures. While the implant is being inserted the micromotor shows again the corresponding torque–depth curve (**b**), together with the Cm, Cp (IT), I and d values. After implants were inserted their stability was measured through Resonance Frequency Analysis (RFA) (**c**) and reverse torque (RT) measurement (**d**).

The density of each bone block was measured as follows. A narrow 14 mm-deep hole was prepared using a 2.2-mm-diameter drill. Depth was controlled using the correspondent drill stopper provided by the drilling set manufacturer (IDI Evolution, Concorezzo, Italy). Subsequently, a dedicated bone density measuring probe was mounted on the micromotor handpiece (Figure 1b) and the first probe thread was inserted in the access hole. The probe was let to screw into the hole at a given speed (30 rpm) (Figure 1c) and the device measured the friction with the bone walls (Figure 1d), a quantity related to, and therefore measuring, bone density. Six out of the fifteen blocks were chosen having density values as equidistant as possible throughout the whole density range, and six implants were inserted in each of them (Figure 2a). Site preparation was performed according to the drilling sequence advised by the implant manufacturer (Table 1). Implants were inserted at a constant speed of 35 rpm, that is, the same to be used in the clinical setting according to the implant manufacturer instruction for implant placement. The first set of six density measurements and six implant insertions in each of the six blocks was carried out in a dry condition. After that, six more bone density measurements and six more implant insertions were carried out in each block under irrigation. Irrigation was carried out using saline, at room temperature, and was external. During insertion, the micromotor displayed the quantities of interest (Figure 2b) that were also recorded in its solid-state memory and then downloaded to a USB key and hence to a personal computer for statistical analysis. After implant placement, Resonance Frequency Analysis (RFA) measurements were performed using an Osstell Mentor device (Osstell AB, Goteborg, Sweden) according to the manufacturer’s instructions (Figure 2c). One experimenter connected the SmartPeg (Osstell AB) to the implant using a manual wrench. The connection torque was 5 Ncm. Two ISQ measurements were then collected along 2 orthogonal directions, aiming the device probe at the SmartPeg. RT measurements (Figure 2d) were performed connecting a high-precision manual dynamometer (ATG6CN Torque Gauge; Tohnichi Mfg Co., Tokyo, Japan) to the implant and applying a counterclockwise torque. When the initial unscrewing of the implant occurred, an independent experimenter read the display, showing the correspondent RT value. 

### 2.5. Data Analysis

Scatter plots were created showing density measurements recorded by the micromotor as a function of actual bone block density, and the correlation between the two density measurements was investigated by means of a linear regression analysis. The relation between density and irrigation was investigated calculating the ratio between the averaged mean torque values collected with and without irrigation. A regression analysis was then carried out between the resulting values and the actual density of bone blocks. To assess the variation of the torque–depth integral within the density range under examination, a linear regression analysis was performed on the integral–density plot. IT-density, RT-density, and ISQ-density were also drawn, and the relation between each implant stability measuring parameter and density was again investigated by means of linear regression analysis. The relation between the integral and the other primary stability parameters (IT, ISQ, and RT) was investigated through a correlation analysis and calculating the corresponding Spearman r coefficients.

## 3. Results

Blocks used in the present study had the density showed in Table 1.

**Figure 3:**
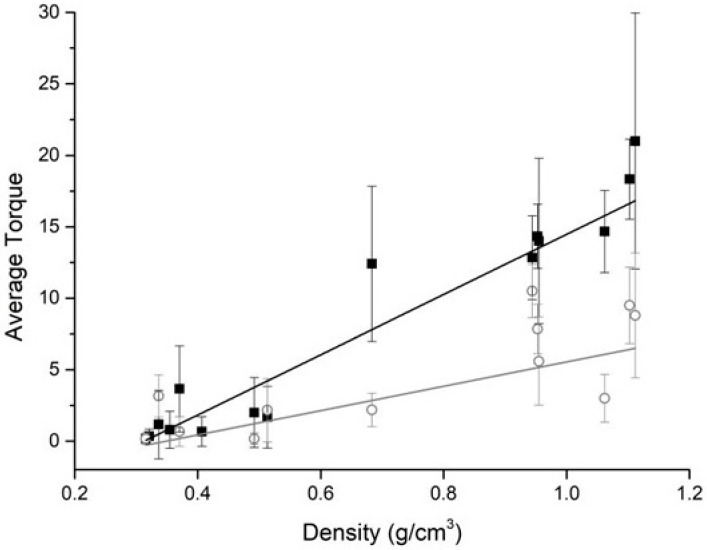
Plots of the mean torque (N × cm) measured by the instrument and the actual density of the bone blocks. Graphs obtained plotting average torque values collected without irrigation (black squares) and with irrigation (gray circles) are shown.

Scatter plots showing how bone density measurements recorded using the micromotor varied as a function of the actual block density are shown in Figure 3.

**Figure 4:**
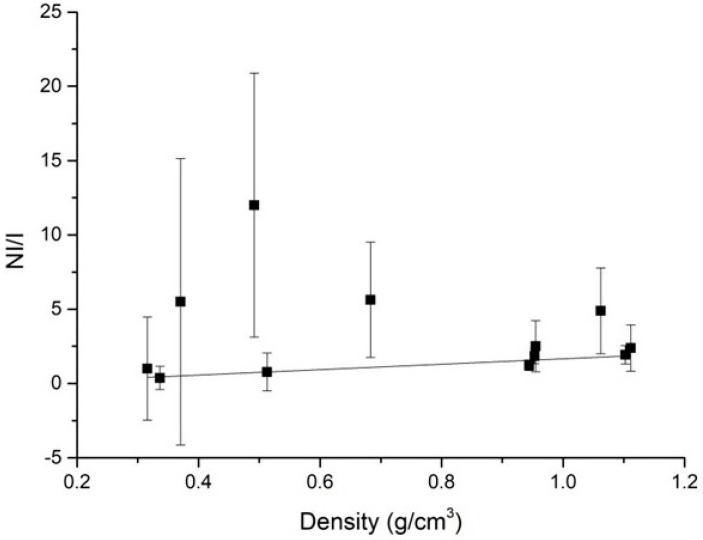
Plot of the ratio between bone-density values measured without irrigation (NI) and under irrigation (I) versus the actual density of the equine bone blocks.

The relation between the two quantities was found to be linear (under irrigation, Pearson’s r = 0.764; without irrigation, r = 0.977), and the two equations (y = mx + q) of the lines that best fitted the experimental points, achieved by regression analysis, are as follows; with irrigation, m = 8.52 ± 2.27; q = –2.97 ± 1.14; without irrigation, m = 21.06 ± 1.27; q = –6.59 ± 0.50. Figure 4 shows how the ratio between the averaged mean torque values collected with irrigation and without irrigation varied with density, the regression analysis showing a weak correlation (r = 0.559), and the ratio increasing with density according to a linear equation (y = mx + q), having coefficients m = 1.82 ± 0.86; q = –0.17 ± 0.79.

**Figure 5:**
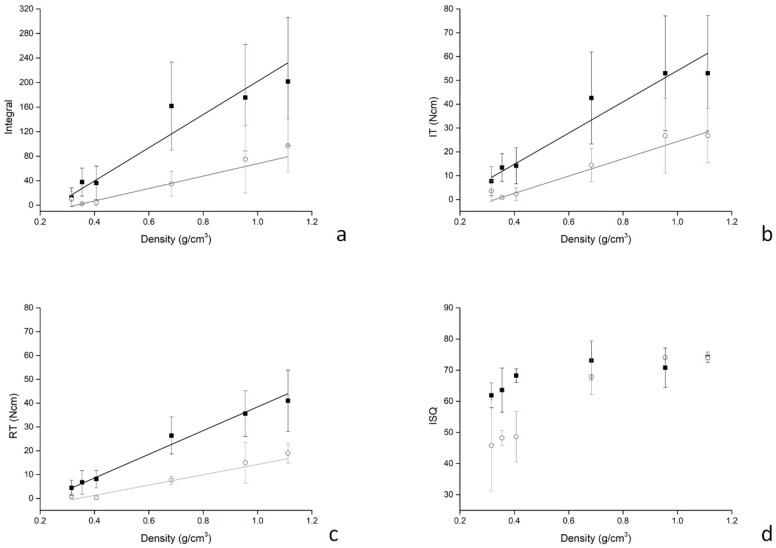
Plots showing how primary stability measurements, that is the (**a**) torque–depth curve integral, (**b**) the insertion torque (IT), (**c**) the reverse torque (RT), and (**d**) the implant stability quotient (ISQ) varied with density. Superimposed lines are those achieved by linear regression analysis. The slope of the integral–density curve (see values on y-axis in plot (a)) is greater than those of IT-density and RT-density, indicating that the integral is more sensitive to density variation than the other two parameters. The ISQ-density plot shows an altogether different shape (see text for comment).

Integral–density plots are shown in Figure 5a. Regression analysis showed the relation between the two quantities to be linear both under irrigation (r = 0.921) and without irrigation (Pearson’s r = 0.959), the equations (y = mx + q) of the lines best fitting the experimental points being with irrigation, m = 101.24 ± 21.35; q = –33.42 ± 7.66; without irrigation, m = 270.40 ± 40.07; q = –68.49 ± 15.62. IT-density, RT-density plots are shown in Figure 5b,c. For both IT and RT, their relations with density was found to be linear (IT, under irrigation: r = 0.957, m = 36.26 ± 5.47; q = –11.92 ± 2.02; without irrigation: r = 0.968, m = 65.52 ± 8.38; q = –11.42 ± 3.53; RT, under irrigation: r = 0.941, m = 21.61 ± 4.49; q = –7.36 ± 2.03; without irrigation: r = 0.992, m = 49.73 ± 3.17; q = –11.31 ± 1.46). ISQ-density plots (Figure 5d) had a quite different shape, with ISQ values being different when collected with or without irrigation at lower densities but overlapping at higher densities.

Results of correlation analysis between the integral, IT, RT, and ISQ values collected with and without irrigation are provided in Table 2. The torque–depth integral was found to correlate significantly with IT and RT both when implants were inserted with and without irrigation (*p* < 0.01 in all cases). It correlated significantly with ISQ only when implants were inserted without irrigation (*p* = 0.02). While ISQ correlated significantly with all other implant stability parameters when implants were inserted without irrigation (*p* < 0.05 in all cases), adding irrigation led to a loss of such correlation, that could be still observed only between ISQ and IT, even if nearly not significant (*p* = 0.049).

## 4. Discussion

Results of the present study show that the average torque that a bone probe exerts to screw into a narrow, predrilled tunnel at the implant placement site at a constant speed is a good estimator of the bone density at that site. These findings are consistent with those of the authors that first introduced the idea of using friction at drilling as an estimator of bone density [37,38], and with previous findings on polyurethane foam blocks [17]. The calibration curve drawn in the present study, though, presents a quite different slope, both with and without irrigation, from that observed when foam blocks were used [17]. In that study, in fact, the slope of the line best fitting the average torque-density plot was m = 33.95 ± 4.93 under irrigation and m = 43.97 ± 1.46 without irrigation; in this study, the corresponding observed slopes are m = 8.52 ± 2.27 with irrigation and m = 21.06 ± 1.27 without irrigation, the ratio (foam block/bone block) being 3.98 and 2.09, respectively. Such observations indicate that (a) equine bone blocks having the same density of foam blocks exert a significantly smaller friction on the probe used to measure bone density than foam blocks and (b) that irrigation in bone blocks leads to a greater decrease in friction than that observed in foam blocks. Given the structure and composition of the two experimental systems, the foam block being made of solid plastic, bone blocks being made of bone apatite having a natural trabecular structure, it looks reasonable to suppose that the calibration curves drawn in the present study may better approximate the condition found in the clinical setting when using the micromotor to measure the site-specific bone density at the implant placement site. In this study, the lubricating effect of irrigation was not found to be dependent on bone density as it was in the investigation using foam blocks; again, this might be due to the substantial differences in composition and structure of the two experimental systems [27]. Results of the present study concerning the integral of the torque–depth curve confirm that such parameter is a good estimator of the implant primary stability as already observed in foam blocks and that the integral displays a greater sensitivity to density variation than IT and RT [25]. In this study, though, the integral–density curves did not display a plateau at higher densities as they did in foam blocks, indicating that high density equine bone blocks behave differently from high density foam blocks as far as the energy exchange with the implant is concerned. When the implant threads are evenly spaced and the implant is placed at a constant rotation speed, two conditions met in the present study, the integral of the torque–depth curve measures the total energy that is needed to place the implant into its seat. Such energy is dispersed as heat caused by friction and into the elastic and anelastic deformations of the material surrounding the implant; the lack of a plateau in the integral–density curves observed in the present study indicates that the elastic and anelastic response of bone is substantially different than that of foam, probably because of the difference in structure and composition of the two materials. Observations of the present study confirm that the integral of the torque–depth curve, the IT and the RT provide more information about, or are more affected by, the fixture resistance to shear forces than to bending forces. Such difference, already suggested by Sennerby et al. [39] on a theoretical basis and by results of a past study on foam blocks [25] seems supported by the results of the present study, showing an ISQ-density plot having a different shape than the integral-, IT- and RT-density ones. The ISQ-density plot, in fact, shows a marked plateau, with ISQ values collected with and without irrigation overlapping at higher densities, consistent with a model implying that at higher densities, the presence of liquid at the bone–implant interface may work less effectively as a cushion to lateral vibrations than at lower densities, where it may be more effective in slowing down the frequency of lateral dislodgement. The substantial lack of correlation that was observed in the present study between the integral, IT and RT when measurements were collected under irrigation might again be explained by the ISQ parameter having a different mechanical meaning from the other stability parameters: it is reasonable to suppose that irrigation may affect the integral, IT and RT differently from ISQ, as their values depend on the friction at rotation, and not on lateral dislodgement. Finally, the results of the present study show that the experimental model that has been used, equine collagen-preserving bone blocks, allows consistent implant placement, without breaking, in a material that should be, by nature, much more similar to human bone than foam blocks. The main contribution of this work is therefore that of showing that results that were observed on a synthetic bone model also hold on a model closer to human bone, indicating that bone density and implant stability measurements, if collected in the clinical setting, might be actually used by the oral surgeon to diagnose bone quality and plan implant insertion appropriately.

Yet, applicability of results of the present study to human bone should further be tested, possibly using homologous (tissue bank) bone. In general, further studies should be aimed to characterize these blocks as a testing material and investigate their reliability in reproducing human alveolar bone mechanical properties consistently. This study has other main limitations. One is that even when implant insertion is carried out under irrigation, this condition mimics only partially that being encountered in skeletal bone, where a constant blood flow is present; second, the number of density measurements and implants insertions carried out in this study was relatively small; further, bone blocks used in the present study were made of cancellous bone only, and had no cortical layer; and finally, different implant geometries and surfaces might produce significantly different results. Results of the present study cannot therefore be generalized, and each of these limitations should be addressed carrying out specific, targeted studies.

## 5. Conclusions

A probe that senses the friction encountered while screwing in the implant tunnel provides a site-specific, reliable measurement of bone density. The integral of the torque–depth curve at implant insertion is a reliable estimator of primary implant stability, more sensitive to density variations than IT, RT, and ISQ. Equine cancellous bone blocks display some characteristics that might make them useful as a testing material to investigate implant stability. Results of this study call for further investigations.

## Figures and Tables

**Table 1:** Density of bone blocks used in the present study and the last drill sequence used to prepare blocks implants were inserted into.

Block	Density (g/cm^3^)	Body Preparation (∅, mm)	Head Preparation (∅, mm)
1	0.316 ± 0.003	2.8	3.7
2	0.321 ± 0.005	-	-
3	0.337 ± 0.003	-	-
4	0.355 ± 0.003	2.8	3.7
5	0.371 ± 0.006	-	-
6	0.407 ± 0.006	2.8	3.7
7	0.492 ± 0.008	-	-
8	0.513 ± 0.006	-	-
9	0.683 ± 0.203	3.2	3.7
10	0.944 ± 0.114	-	-
11	0.953 ± 0.068	-	-
12	0.955 ± 0.072	3.4	3.7
13	1.062 ± 0.062	-	-
14	1.102 ± 0.115	-	-
15	1.112 ± 0.044	3.4	4.0

**Table 2:** Spearman’s r coefficients measuring the correlation between the integral (I), the insertion torque (IT), the reverse torque (RT) and the implant stability quotient (ISQ) recorded at implant placement without irrigation (above the diagonal) and with irrigation (below the diagonal). Significant values are marked with an asterisk (*) and statistical significance is shown in italic.

	I	IT	RT	ISQ
I	1	0.9276 *	0.9429 *	0.8857 *
	-	*0.008*	*0.005*	*0.019*
IT	0.9861 *	1	0.9856 *	0.8986 *
	*<0.001*	-	*<0.001*	*0.014*
RT	0.9973 *	0.9856 *	1	0.9429 *
	*<0.001*	*<0.001*	-	*0.005*
ISQ	0.7714	0.8117 *	0.77143	1
	*0.072*	*0.049*	*0.072*	-

## References

[1] Marquezan M., Osorio A., Sant’Anna E., Souza M.M., Maia L. (2012). Does bone mineral density influence the primary stability of dental implants? A systematic review. Clin. Oral Implants Res..

[2] Molly L. (2006). Bone density and primary stability in implant therapy. Clin. Oral Implants Res..

[3] Javed F., Ahmed H.B., Crespi R., Romanos G.E. (2013). Role of primary stability for successful osseointegration of dental implants: Factors of influence and evaluation. Interv. Med. Appl. Sci..

[4] Javed F., Romanos G.E. (2010). The role of primary stability for successful immediate loading of dental implants. A literature review. J. Dent..

[5] Howashi M., Tsukiyama Y., Ayukawa Y., Isoda-Akizuki K., Kihara M., Imai Y., Sogo M., Koyano K. (2016). Relationship between the CT Value and Cortical Bone Thickness at Implant Recipient Sites and Primary Implant Stability with Comparison of Different Implant Types. Clin. Implant Dent. Relat. Res..

[6] Hong J., Lim Y.J., Park S.O. (2012). Quantitative biomechanical analysis of the influence of the cortical bone and implant length on primary stability. Clin. Oral. Implants Res..

[7] Morgan E.F., Bouxsein M.L., Bilezikian P.J., Raisz L.G., Martin J.T. (2008). Bone Biomechanics. Principles of Bone Biology.

[8] Wallace M.J., Burr D.B., Allen M.R. (2019). Skeletal Hard Tissue Biomechanics. Basic and Applied Bone Biology.

[9] Robertson D.M., Smith D.C. (1978). Compressive strength of mandibular bone as a function of microstructure and strain rate. J. Biomech..

[10] Schaffler M.B., Burr D.B. (1988). Stiffness of compact bone: Effects of porosity and density. J. Biomech..

[11] Rho J.Y., Kuhn-Spearing L., Zioupos P. (1998). Mechanical properties and the hierarchical structure of bone. Med. Eng. Phys..

[12] Ko Y.C., Huang H.L., Shen Y.W., Cai J.Y., Fuh L.J., Hsu J.T. (2017). Variations in crestal cortical bone thickness at dental implant sites in different regions of the jawbone. Clin. Implant Dent. Relat. Res..

[13] Wang R., Eppell S.J., Nguyen C., Morris N. (2016). Relative Contribution of Trabecular and Cortical Bone to Primary Implant Stability: An In Vitro Model Study. J. Oral Implantol..

[14] De Oliveira Nicolau Mantovani A.K., de Mattias Sartori I.A., Azevedo-Alanis L.R., Tiossi R., Fontão F.N.G.K. (2018). Influence of cortical bone anchorage on the primary stability of dental implants. Oral Maxillofac Surg..

[15] Degidi M., Daprile G., Piattelli A. (2015). Influence of underpreparation on primary stability of implants inserted in poor quality bone sites: An in vitro study. J. Oral Maxillofac. Surg..

[16] Iezzi G., Scarano A., Di Stefano D.A., Arosio P., Doi K., Ricci L., Piattelli A., Perrotti V. (2015). Correlation between the bone density recorded by a computerized implant motor and by a histomorphometric analysis: A preliminary in vitro study on bovine ribs. Clin. Implant Dent. Relat. Res..

[17] Di Stefano D.A., Arosio P. (2016). Correlation between Bone Density and Instantaneous Torque at Implant Site Preparation: A Validation on Polyurethane Foam Blocks of a Device Assessing Density of Jawbones. Int. J. Oral Maxillofac. Implants.

[18] Degidi M., Daprile G., Piattelli A., Iezzi G. (2013). Development of a new implant primary stability parameter: Insertion torque revisited. Clin. Implant Dent. Relat. Res..

[19] Kim S.H., Lee S.J., Cho I.S., Kim S.K., Kim T.W. (2009). Rotational resistance of surface-treated mini-implants. Angle Orthod..

[20] Park K.J., Kwon J.Y., Kim S.K., Heo S.J., Koak J.Y., Lee J.H., Lee S.J., Kim T.H., Kim M.J. (2012). The relationship between implant stability quotient values and implant insertion variables: A clinical study. J. Oral Rehabil..

[21] Iezzi G., Filippone A., Stefano D.A., Arosio P., Piattelli A., Scarano A., Perrotti V. (2015). A site-specific intraoperative measurement of bone-to-implant contact during implant insertion: A study on bovine ribs using a computerized implant motor. J. Dent. Sci..

[22] Cappare P., Vinci R., Di Stefano D.A., Traini T., Pantaleo G., Gherlone E.F., Gastaldi G. (2015). Correlation between Initial BIC and the Insertion Torque/Depth Integral Recorded with an Instantaneous Torque-Measuring Implant Motor: An in vivo Study. Clin. Implant Dent. Relat. Res..

[23] Arosio P., Greco G.B., Zaniol T., Iezzi G., Perrotti V., Di Stefano D.A. (2018). Sinus augmentation and concomitant implant placement in low bone-density sites. A retrospective study on an undersized drilling protocol and primary stability. Clin. Implant Dent. Relat. Res..

[24] Degidi M., Daprile G., Piattelli A. (2017). Influence of Stepped Osteotomy on Primary Stability of Implants Inserted in Low-Density Bone Sites: An In Vitro Study. Int. J. Oral Maxillofac. Implant.

[25] Di Stefano D.A., Arosio P., Gastaldi G., Gherlone E. (2018). The insertion torque-depth curve integral as a measure of implant primary stability: An in vitro study on polyurethane foam blocks. J. Prosthet. Dent..

[26] ASTM F1839-08(2016) (2016). Standard Specification for Rigid Polyurethane Foam for Use as a Standard Material for Testing Orthopaedic Devices and Instruments.

[27] Grant J.A., Bishop N.E., Gotzen N., Sprecher C., Honl M., Morlock M.M. (2007). Artificial composite bone as a model of human trabecular bone: The implant-bone interface. J. Biomech..

[28] De Angelis N., Scivetti M. (2011). Lateral ridge augmentation using an equine flex bone block infused with recombinant human platelet-derived growth factor BB: A clinical and histologic study. Int. J. Periodontics Restor. Dent..

[29] Di Stefano D.A., Gastaldi G., Vinci R., Cinci L., Pieri L., Gherlone E. (2015). Histomorphometric Comparison of Enzyme-Deantigenic Equine Bone and Anorganic Bovine Bone in Sinus Augmentation: A Randomized Clinical Trial with 3-Year Follow-Up. Int. J. Oral Maxillofac. Implants.

[30] Di Stefano D.A., Gastaldi G., Vinci R., Polizzi E.M., Cinci L., Pieri L., Gherlone E. (2016). Bone Formation Following Sinus Augmentation with an Equine-Derived Bone Graft: A Retrospective Histologic and Histomorphometric Study with 36-Month Follow-up. Int. J. Oral Maxillofac. Implants.

[31] Eser C., Gencel E., Gokdogan M., Kesiktas E., Yavuz M. (2015). Comparison of autologous and heterologous bone graft stability effects for filling maxillary bone gap after Le Fort I osteotomy. Adv. Clin. Exp. Med..

[32] Iorio R., Pagnottelli M., Vadala A., Giannetti S., Di Sette P., Papandrea P., Conteduca F., Ferretti A. (2013). Open-wedge high tibial osteotomy: Comparison between manual and computer-assisted techniques. Knee Surg. Sports Traumatol. Arthrosc..

[33] Mattioli B., Iacoviello P., Aldiano C., Verrina G. (2018). Subcranial Le Fort III Advancement with Equine-Derived Bone Grafts to Correct Syndromic Midfacial Hypoplasia: A Case Report. J. Maxillofac. Oral Surg..

[34] Pistilli R., Signorini L., Pisacane A., Lizio G., Felice P. (2013). Case of severe bone atrophy of the posterior maxilla rehabilitated with blocks of equine origin bone: Histological results. Implant Dent..

[35] Santini S., Barbera P., Modena M., Schiavon R., Bonato M. (2011). Equine-derived bone substitutes in orthopedics and traumatology: authors’ experience. Minerva Chir..

[36] Sonmez M.M., Armagan R., Ugurlar M., Eren T. (2017). Allografts versus Equine Xenografts in Calcaneal Fracture Repair. J. Foot Ankle Surg..

[37] Friberg B., Sennerby L., Roos J., Johansson P., Strid C.G., Lekholm U. (1995). Evaluation of bone density using cutting resistance measurements and microradiography: An in vitro study in pig ribs. Clin. Oral Implants Res..

[38] Friberg B., Sennerby L., Roos J., Lekholm U. (1995). Identification of bone quality in conjunction with insertion of titanium implants. A pilot study in jaw autopsy specimens. Clin. Oral Implants Res..

[39] Sennerby L., Meredith N. (2008). Implant stability measurements using resonance frequency analysis: Biological and biomechanical aspects and clinical implications. Periodontol 2000.

